# Tumor-associated macrophages promote resistance of hepatocellular carcinoma cells against sorafenib by activating CXCR2 signaling

**DOI:** 10.1186/s12929-022-00881-4

**Published:** 2022-11-21

**Authors:** Hao-Chen Wang, Lin-Ya Haung, Chih-Jung Wang, Ying-Jui Chao, Ya-Chin Hou, Chia-Jui Yen, Yan-Shen Shan

**Affiliations:** 1 Institute of Clinical Medicine, College of Medicine, National Cheng Kung University, No. 35, Xiaodong Road, Tainan, 704017 Taiwan; 2 Department of Surgery, National Cheng Kung University Hospital, College of Medicine, National Cheng Kung University, No. 138, Shengli Road, Tainan, 704302 Taiwan; 3 Department of Oncology, National Cheng Kung University Hospital, College of Medicine, National Cheng Kung University, No. 138, Shengli Road, Tainan, 704302 Taiwan

**Keywords:** Hepatocellular carcinoma, Sorafenib resistance, Tumor-associated macrophage, CXCR2, CXCL1, CXCL2

## Abstract

**Background:**

Sorafenib (SOR) is the first line treatment for advanced hepatocellular carcinoma (HCC), but resistance develops frequently. Tumor-associated macrophages (TAMs) have been reported to affect the progression of HCC. We therefore aimed to study the role of TAMs in promoting SOR resistance.

**Methods:**

Immunofluorescence staining for the M2 marker CD204 and the cancer stem cell (CSC) markers CD44 and CD133 was performed in paired HCC and adjacent noncancerous tissues and HCC tissues stratified by response of SOR treatment. HCC/U937 coculture system and cytokines were used to induce M2 polarization for studying the effects of M2 TAMs on CSC properties and apoptotic death of HCC cells after SOR treatment.

**Results:**

Higher expression of CD204, CD44, and CD133 was observed in patients with SOR nonresponse (SNR) than in those with SOR response (SR), suggesting that SNR is positively correlated to levels of CSCs and M2 TAMs. After coculture, M2 TAMs could increase the level of CSCs but decrease SOR-induced apoptosis. Incubation of HCC cells with coculture conditioned medium increased the formation of spheres that were resistant to SOR. Furthermore, CXCL1 and CXCL2 were found to be the potential paracrine factors released by M2 TAMs to upregulate SOR resistance in HCC cells. Treatment with CXCL1 and CXCL2 could increase HCC CSC activity but decrease SOR-induced apoptosis by affecting BCL-2 family gene expression. Using pharmacological inhibitors, CXCR2/ERK signaling was found to be critical to CXCL1- and CXCL2-mediated SOR resistance.

**Conclusion:**

This study identified CXCL1, CXCL2, and their downstream CXCR2/ERK signaling as potential therapeutic targets to overcome SOR resistance in HCC.

**Supplementary Information:**

The online version contains supplementary material available at 10.1186/s12929-022-00881-4.

## Background

Hepatocellular carcinoma (HCC) is the sixth most common cancer and the third leading cause of cancer death worldwide [32]. Surgery, liver transplant, and local ablation are the most effective treatment modalities to achieve cure for HCC. Unfortunately, many patients are not suitable for these treatments because of large tumor size, the invasion into neighboring tissues, or the spreading to different sites [6]. For the treatment of advanced-stage HCC, targeted therapies with tyrosine kinase inhibitors (TKIs) such as sorafenib (SOR) that inhibits VEGFR1, VEGFR2, VEGFR3 and PDGFR-α, PDGFR-β, c-KIT, Raf-1 are recommended [24]. However, low response rates to SOR and short effective duration in clinical trials indicate that drug resistance is a common event [9, 24], which emphasizing the urgent need to combat drug resistance for HCC patients by developing novel therapeutic approaches.

The tumor microenvironment (TME) is composed of immune cells, stromal cells, blood vessels, extracellular matrix, and signaling molecules and has been recognized as a key factor in driving tumor development and progression. Macrophages that infiltrate in the tumor, also called tumor-associated macrophages (TAMs), represent the most abundant immune cells in the TME [27]. The high plasticity of macrophages enables them to alter their phenotype. The classically M1 activated phenotype induced by IFN-γ or lipopolysaccharide exhibits tumoricidal properties. On the other hand, macrophages with the M2 phenotype alternatively activated by IL-4 or IL-10 are endowed with tumor-promoting activities. Compelling evidence has highlighted the association of the high density of M2 TAMs with poor patient outcome in many cancers [2, 19, 28]. Depletion of M2 TAMs in the TME effectively reduced tumor burden [4, 29], suggesting the therapeutic potential of targeting macrophages in the TME for cancer treatment.

Recent research has brought forth the important role of TAMs in mediating acquired drug resistance of tumor cells, and depleting these cells can restore the response to chemotherapy. For instance, paclitaxel treatment increased TAMs in the PyMT mouse model of breast cancer, and these macrophages in turn protected tumor cells from cell death induced by chemotherapeutic drugs [31]. TAMs can secret cytokines such as IL-6 to induce chemoresistance in colorectal cancer cells via the IL6R/STAT3/miR-204-5p pathway [37]. Targeting TAMs with CSF-1 receptor inhibitor could foster antitumor immunity and improve response to cytotoxic therapy in murine models [13, 14]. In this study, we aimed to identify the detailed roles for M2 TAMs in SOR resistance of HCC cells and unravel the underlying molecular mechanisms.

## Methods

### Immunofluorescence (IF) staining of clinical HCC samples

A total of 109 HCC tissues and their matched adjacent peritumoral tissues were collected from patients undergoing surgical resection in National Cheng Kung University Hospital (NCKUH). The clinical records of the HCC patients were retrospectively analyzed in this study approved by Institutional Review Board of NCKUH (IRB No. B-ER-104-258). Anonymous archived samples of human HCC were obtained from Human Biobank of NCKUH. The paraffin-embedded tissue sections were incubated in sodium citrate buffer (10 mM, pH 6.0) and heated by autoclave at 121 °C for 10 min for antigen retrieval. All slides were incubated with primary antibodies at 4 °C overnight followed by Alexa Fluor® conjugated secondary antibodies at room temperature for 1 h. Fluorescence images were visualized and captured with a fluorescence microscope (BX53; Olympus), and the signals were quantified with the Tissue-Quest software (TissueGnostics GmbH).

### Cell culture

The human myeloid leukaemia cell line U937 and the human hepatocellular carcinoma cell lines HepG2, Hep3B, and Huh7 were maintained in RPMI 1640 medium (Hyclone) supplemented with 10% fetal bovine serum (Gibco), 1 × antibiatic-antimycotic (GeneDireX), 200 μM L-Glutamine (GeneDireX), and 1.5 mM HEPES (GeneDireX). All cells were cultured with 5% CO_2_ at 37℃ in humidified atmosphere.

### In vitro coculture assay

HepG2 cells were seeded into cell culture transwell inserts (3 × 10^5^ cells per insert) with pore size of 0.4 µm (Falcon) packed in a 6-well plate (GeneDireX) where U937 cells were grown (3 × 10^5^ cells per well). The cocultures were incubated at 37 °C for 72 h. The coculture conditioned medium (CM) was collected for the generation of M2 macrophages.

### Generation of M2 macrophages

U937 cells were induced to M2 phenotype by treatment with phorbol 12-myristate 13-acetate (PMA; 100 nM; Sigma) for 24 h followed by interleukin-4 (IL-4; 20 ng/mL; Cell Guidance System) in combination with interleukin-10 (IL-10; 20 ng/mL; Cell Guidance System) for 48 h (called M2 Mφs^Cyto^). Alternatively, M2 macrophages were generated by treatment with 10% of HepG2/U937 coculture CM for 48 h (called TAMs^CM^). The cells were stained with FITC anti-human CD68 (BioLegend, #333806) and PE anti-human CD204 (BioLegend, #371904) in 4 °C for 30 min. The M2 phenotype was determined by detecting CD68/CD204 expression using a FACS Calibur flow cytometer (BD Biosciences).

### RNA interference (RNAi) and generation of stable cell lines

*NANOG*, *SOX2*, and *OCT4* short hairpin RNA lentiviral particles were purchased from the National RNAi Core Facility, Academia Sinica (Taipei, Taiwan). HepG2 cells were infected with lentivirus in the presence of polybrene (8 μg/mL; Sigma). Puromycin (5 μg/mL; Sigma) was used for selection of stable transfectants.

### Sphere formation assay

HCC cells were cultured in 6-well ultra-low attachment surface dishes (Corning) at the cell density of 2,000 cells/well with stem-cell medium, serum-free DMEM/F12 (Life Technologies) supplemented with N-2 supplement (Life Technologies), 10 ng/mL recombinant human epithelial growth factor (R&D Systems), 10 ng/mL human basic fibroblast growth factor (R&D Systems). Two weeks after cell seeding, sphere formation was observed under a light microscope. A cell cluster with the diameter longer than 100 µm was regarded as a sphere.

### Cell viability assay

The cytotoxicity of SOR against HCC cells was assessed by the colorimetric 3-(4,5-dimethylthiazol-2-yl)-2,5-diphe-nyltetrazolium bromide (MTT) assay. Cells were seeded in a 96-well plate (GeneDireX) at the density of 2 × 10^3^/well. SOR was added at different concentrations to the wells one day after seeding. Two days after drug treatment, MTT (Sigma) solution was added to the wells at a final concentration of 0.5 mg/mL and incubated at 37℃ for 3 h. After removal of the supernatant, the formazan crystal was dissolved with 100 μL DMSO (Scharlau) and the absorbance was read at 595 nm using a TECAN Sunrise ELISA Reader.

### Cell apoptosis

Cell apoptosis was detected using a FITC Annexin V Apoptosis Detection Kit (BD Biosciences) according to the manufacturer’s protocol. HCC cells treated with or without SOR were harvested and were stained with 5% Annexin V-FITC and 5% propidium iodide (PI) for 15 min at room temperature in dark. Stained cells were detected and analyzed using a FACS Calibur flow cytometer (BD Biosciences).

### Identification of cancer stem cells (CSCs)

To delineate cancer stem cell markers CD44 and CD133 expression in HCC cells, flow cytometry was used to detect the levels of the cell surface proteins. Cells were harvested and incubated with FITC mouse anti-human CD44 (BD PharmingenTM, #555478) and PE anti-human CD133 (BioLegend, #372804) in 4 °C for 30 min. The measurement was performed using a FACS Calibur flow cytometer (BD Biosciences).

### RNA extraction, reverse transcription, and real-time quantitative polymerase chain reaction (qPCR)

Total RNA was extracted using Total RNA Purification Kit (TR01, GeneMark) according to the manufacturer’s instructions. RNA concentration was determined by NanoDrop spectrophotometer (Thermo Fisher Scientific). To reverse transcribe, 1 µg of total RNA was mixed with 0.5 µM of Oligo-dT primer (Invitrogen), 1 × Deoxy + Hispec Reverse Transcripatase premix (Yeastern Biotech), and nuclease-free water to the total volume of 18 µL. After incubation at 65 °C for 5 min, 1 µL of recombinant RNase inhibitor (40 units/µL; Yeastern Biotech) and 1 µL of M-MLV reverse transcriptase (200 units/µL; Promega) was added to the mixture. The mixture was incubated at 30℃ for 10 min, at 42 °C for 60 min, and at 70 °C for 15 min in a thermal cycler (Bio-Rad) to generate first-strand cDNA. The first-strand cDNA was used as a template for qPCR analysis. To perform qPCR, 0.5 µL of cDNA was mixed with 2 µL nuclease-free water, 5 µL 2 × SYBRTM Green master mix (Promega), 1 µL forward primer (finally 1 µM), and 1 µL reverse primer (finally 1 µM), and the reaction was run at the Applied Biosystems StepOneTM (Applied Biosystems) under the following conditions: 95 °C for 5 min followed by 50 cycles of denaturation at 95 °C for 10 s, annealing at 60 °C for 10 s, and extension at 65 °C for 10 s in each. The human *GAPDH* gene was used as an endogenous control in normalizing RNA expression of target genes.

### Total protein extraction and Western blotting

Cells were collected and lysed using RIPA lysis buffer (Millipore) comtaining protease inhibitors (Sigma-Aldrich) at 4 °C for 30 min. The lysate samples were centrifuged at 14,000 rpm for 15 min at 4 °C, and the supernatant was then transferred to a 1.5 mL microcentrifuge tube. Protein extracts were stored at -80℃, and protein concentration was measured by BCA protein assay kit (Thermo). Protein samples for Western blotting were first heated at 95 °C for 5 min. Proteins (30 μg) were separated by SDS–polyacrylamide gel electrophoresis and were then transferred to polyvinylidene difluoride membrane for 100 min at 100 V. The membrane was blocked with 5% bovine serum albumin (BSA) in TBST for 1 h at room temperature followed by washing with TBST thrice and incubation with primary antibodies at 4℃ overnight. After discarding the primary antibody solution, the membrane was washed three times with TBST and incubated with HRP-conjugated secondary antibodies for 1 h at room temperature. The blot signals were developed using Immobilon Western Chemiluminescent HRP Substrate (Millipore) and captured by iBright (Invitrogen).

### Statistical analysis

All statistical analysis was performed using the GraphPad Prism Software (version 5.0). Data were presented as mean ± SEM. Statistical comparison was conducted with Student’s t-test or ANOVA. P value less than 0.05 was considered significant.

## Results

### SOR resistance is positively associated with the levels of M2 TAMs and CSCs in HCC patients

**Fig. 1:**
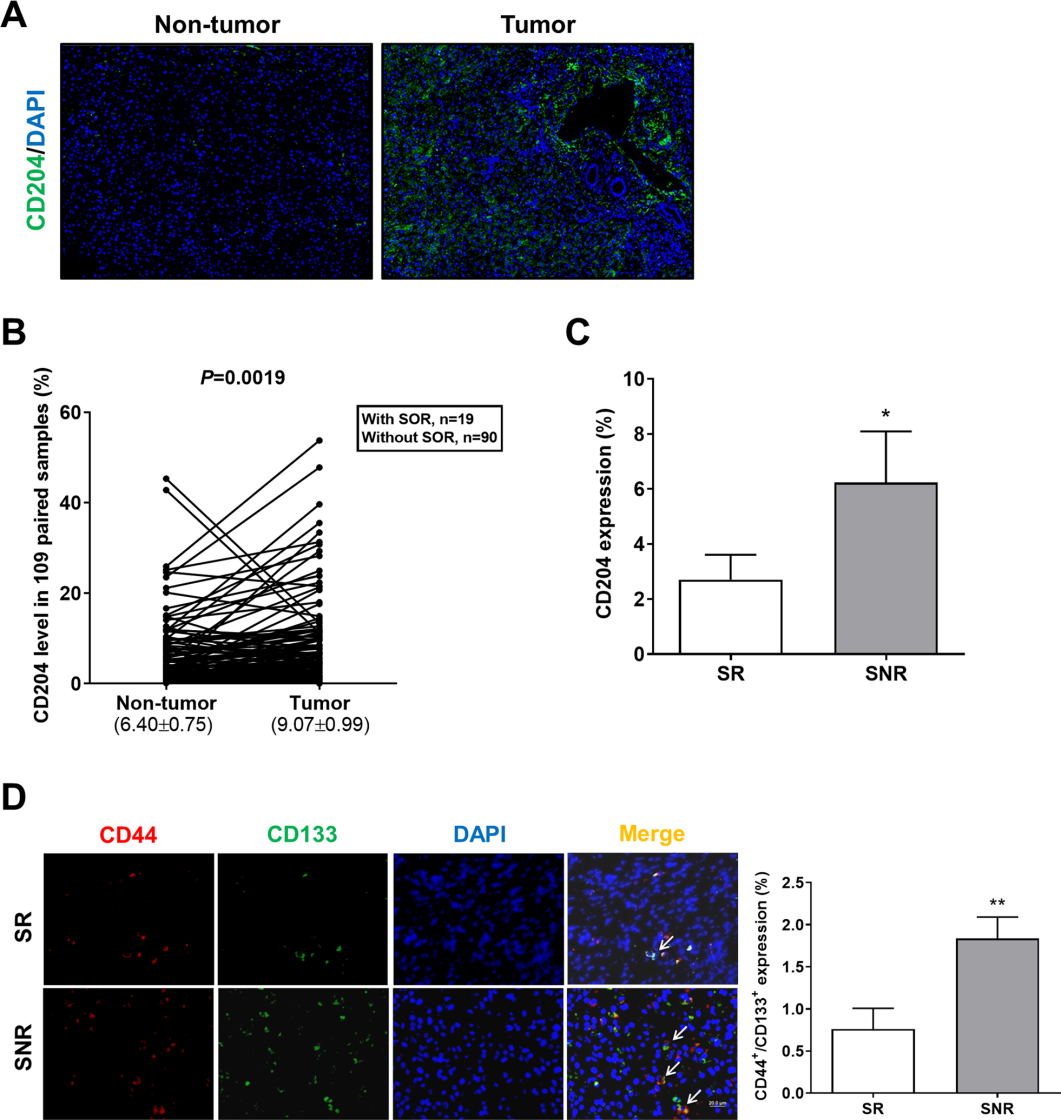
SOR-resistant HCC exhibits increased levels of M2 TAMs and CSCs. **A** Tumor tissue and corresponding non-tumor tissue of HCC were stained for CD204 (green). The staining was visualized by IF microscopy. Magnification: 200 × , scale bar: 20 μm. **B** Paired samples of tumor and normal liver tissue from 109 HCC patients containing 19 with SOR and 90 without SOR were subjected to IF staining for CD204. The results were quantified and represented on the dot plot graph. **C** The bar graph depicts the percentage of CD204 expression in tumor tissues of the SR patients and SNR patients measured by IF staining. *P < 0.05 vs. the SR group. **D** Tumor tissues of SR HCC and SNR HCC were double stained for CD44 (red) and CD133 (green), and the staining was visualized by IF microscopy. White arrows indicate CD44^+^/CD133^+^ HCC cells. Magnification: 200 × , scale bar: 20 μm. The bar graph depicts the percentage of CD44^+^/CD133^+^ expression in tumor tissues of the SR patients and SNR patients

To investigate the role of M2 TAMs in SOR resistance of HCC cells, clinical tumor tissue samples were collected to assess the correlation between the level of M2 TAMs and the patient’s response to SOR. Tissue IF imaging showed higher expression of the M2 marker CD204 in tumor tissue than in matched tumor-adjacent tissue (Fig. 1A). After the quantification of staining positive for CD204, the percentage of positive cell area was higher in tumor tissue than in nontumor tissue (Fig. 1B). We next divided HCC patients receiving SOR into the SOR responsive group (SR, treatment duration ≥ 3 months) and the SOR nonresponsive group (SNR, treatment duration < 3 months). SNR patients exhibited higher CD204 expression in tumor tissue compared with SR patients (Fig. 1C). Given that drug resistance is highly attributed to the presence of CSCs [3] and M2 TAMs can enable cancer cells to acquire CSC properties [8], the level of CSCs was compared between SR HCC and SNR HCC. The CSC surface markers of HCC [40], CD44 and CD133, were expressed more strongly in SNR HCC than in SR HCC (Fig. 1D). Taken together, our results suggest that the resistance to SOR in HCC may result from high infiltration of M2 TAMs and increased cancer stemness.

### M2 TAMs inhibits SOR-induced cytotoxicity in HCC cells

**Fig. 2:**
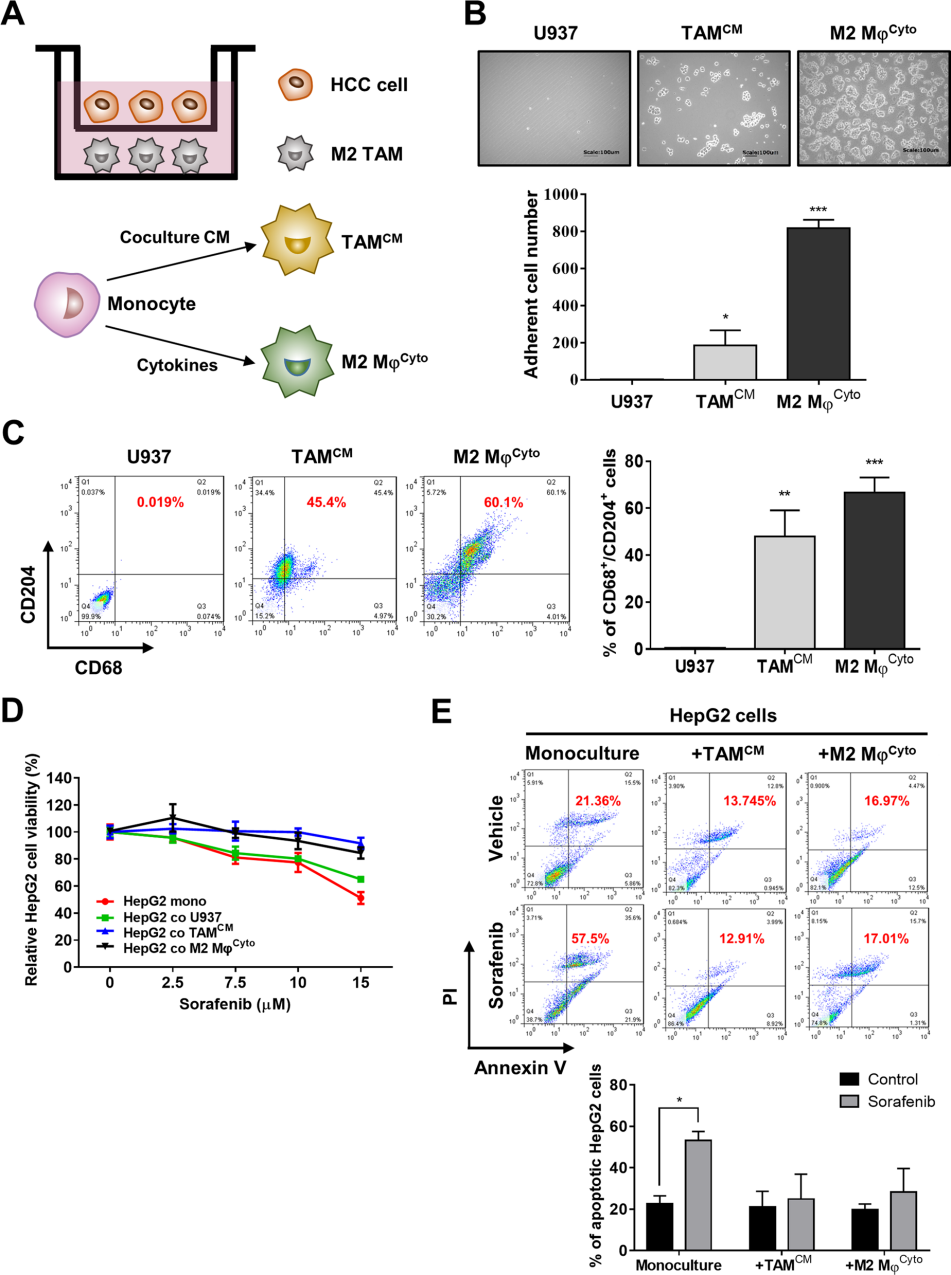
M2 TAMs reduce the cytotoxic effects of SOR on HCC cells. **A** A schematic diagram of transwell HCC cells/M2 TAMs coculture is shown. TAMs^CM^ were generated from U937 cells treated with HCC cells/U937 cells coculture CM for 48 h, and M2 Mφs^Cyto^ were generated from U937 cells treated with PMA for 24 h followed by IL-4 and IL-10 for 48 h. **B** After the differentiation from U937 cells into TAMs^CM^ or M2 Mφs^Cyto^, cell adhesion in the three cells was observed by bright field microscopy. Magnification: 200 × , scale bar: 100 μm. The bar graph depicts the numbers of adherent cells (mean ± SEM of 5 randomly chosen fields of view). *P < 0.05; ***P < 0.001 vs. U937 cells. **C** After differentiation, U937 cells, TAMs^CM^, and M2 Mφs^Cyto^ were collected and subjected to flow cytometry with double staining for CD68 and CD204. The bar graph depicts the percentages of CD68^+^/CD204^+^ cells. **P < 0.01; ***P < 0.001 vs. U937 cells. **D** After monoculture or coculture with TAMs^CM^ or M2 Mφs^Cyto^ for 72 h, HepG2 cells were treated with SOR at different concentrations for 48 h. Cell viability was assessed by MTT assay. **E** After monoculture or coculture with TAMs^CM^ or M2 Mφs^Cyto^ for 72 h, HepG2 cells were treated with SOR (10 μM) for 48 h, and then apoptosis was measured by flow cytometry with double staining for Annexin V and PI. The bar graphs depict the percentages of apoptotic HepG2 cells. *P < 0.05 vs. control

The human monocytic U937 cells can be differentiated towards the M2 phenotype by treatment with Th2 cytokines or by coculture with cancer cells [5, 34]. We have found that, after 72 h of coculture, HepG2 cells could induce the polarization of U937 cells into CD204^+^/CD68^+^ M2 TAMs though the percentage was not high (Additional file 1: Fig. S1). To explore the effect of TAMs on cancer cell behaviors, we generated M2 macrophages from U937 cells by treatment with IL-4/IL-10 and HCC cells/U937 cells coculture CM, named M2 Mφs^Cyto^ and TAMs^CM^, respectively (Fig. 2A). In these two cells, we could see macrophage activation characterized by cell attachment under microscope (Fig. 2B) and increased percentages of macrophages with the M2 phenotype (CD204^+^/CD68^+^) measured by flow cytometry (Fig. 2C). The nonadherent cells that were not polarized were subjected to trypan blue staining, and the images showed that almost all the nonadherent cells were live cells without trypan blue penetration (Additional file 1: Fig. S2A). In addition, cell death and cell viability of U937 cells after incubation with 10% of coculture CM for 48 h were similar to that of U937 cells after incubation with normal medium for 48 h, indicating that our treatments for polarization did not result in toxicity (Additional file 1: Fig. S2B). HepG2 cells were monocultured or cocultured with U937 cells, TAMs^CM^, and M2 Mφs^Cyto^ for 72 h followed by treatment with SOR at different concentrations. The MTT assay demonstrated that, in the presence of SOR, HepG2 cells cocultured with TAMs^CM^, and M2 Mφs^Cyto^ showed higher cell viability than HepG2 monoculture and HepG2 cells cocultured with U937 cells (Fig. 2D). Furthermore, the results of flow cytometry analysis showed that, after treatment with SOR, the rates of cell apoptosis in HepG2 cells cocultured with TAMs^CM^ and M2 Mφs^Cyto^ were 13.75% and 16.97% respectively, lower than that (21.36%) in HepG2 monoculture (Fig. 2E). Taken together, these data reveal an important role for M2 TAMs in sorafenib resistance of HCC cells.

### CSC activity in HepG2 cells is enhanced by M2 TAMs

**Fig. 3:**
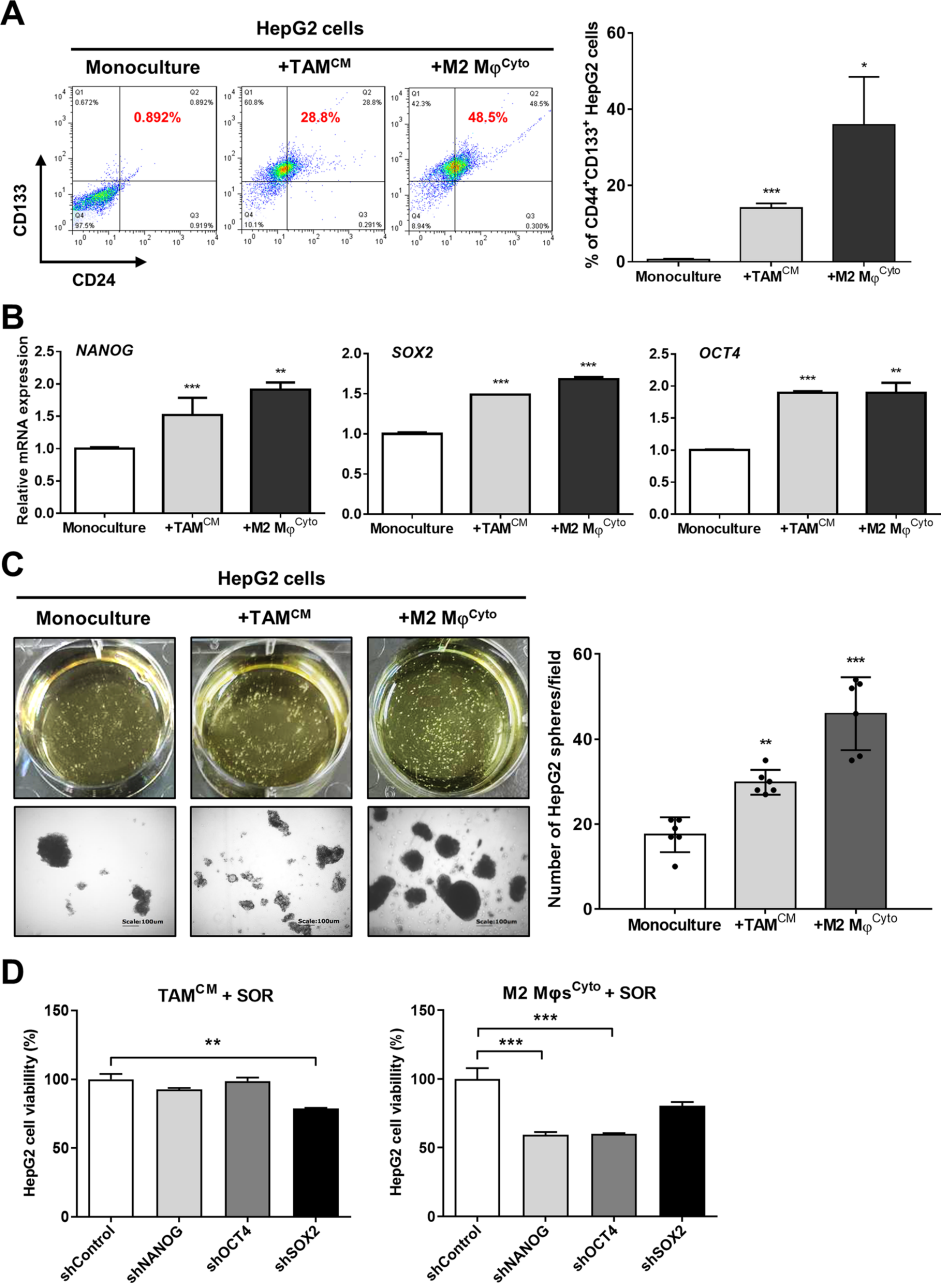
M2 TAMs increase CSC properties in HCC cells. **A** After monoculture or coculture with TAMs^CM^ or M2 Mφs^Cyto^ for 72 h, HepG2 cells were collected and subjected to flow cytometry with double staining for CD44 and CD133. The bar graph depicts the percentages of CD44^+^/CD133^+^ HepG2 cells. *P < 0.05; ***P < 0.001 vs. monoculture. **B** Gene expression of the stem cell markers NANOG, SOX2, and OCT4 in HepG2 cells at 72 h after monoculture or coculture was determined by qPCR. The bar graphs depict the relative mRNA expression. **P < 0.01; ***P < 0.001 vs. monoculture. **C** After monoculture or coculture with TAMs^CM^ or M2 Mφs^Cyto^ for 72 h, HepG2 cells were cultured in ultra-low attachment surface dishes for 14 days to form spheres. The morphology of HepG2 spheres was observed with bright field microscopy (left lower). Magnification, 200 × , scale bar, 100 μm. The bar graph depicts the numbers of HepG2 spheres with a diameter greater than 100 μm in each field. **D** After coculture with TAMs^CM^ or M2 Mφs^Cyto^ for 72 h, HepG2 cells with shControl, shNANOG, shSOX2, or shOCT4 were treated with SOR (10 μM) for 48 h. Cell viability was assessed by MTT assay. The bar graphs depict the relative viability of HepG2 cells. **P < 0.01; ***P < 0.001 vs. shControl

Because CSCs have been considered to underlie cancer therapy resistance, the effect of M2 TAMs on the stemness of HCC cells was investigated. Flow cytometry showed that coculture with TAMs^CM^ and M2 Mφs^Cyto^ increased the average percentages of CD44^+^/CD133^+^ HepG2 cells to 14.1% and 35.9% respectively as compared with 0.6% in HepG2 monoculture (Fig. 3A). Similarly, gene expression of other cancer stemness markers *NANOG*, *SOX2*, and *OCT4* in HepG2 cells was also increased significantly (Fig. 3B). Furthermore, using the sphere formation assay to examine the capacity of CSCs for self-renewal in HCC cells, we found that the numbers of HepG2 spheres were increased to 29.8 and 46 after cocultured with TAMs^CM^ and M2 Mφs^Cyto^, higher than 17.5 spheres in HepG2 monoculture (Fig. 3C). To determine M2 TAMs-induced stemness is responsible for sorafenib resistance, we knocked down the stemness genes *NANOG*, *SOX2*, and *OCT4* in HepG2 cells using RNAi. We found that, after coculture with TAMs^CM^ and M2 Mφs^Cyto^, depletion of *NANOG*, *SOX2*, and *OCT4* in HepG2 cells reversed M2 TAMs-induced sorafenib resistance (Fig. 3D). Taken together, these results suggest that M2 TAMs have ability to enhance CSC characteristics in HCC cells, resulting in enhanced resistance against sorafenib.

**Fig. 4:**
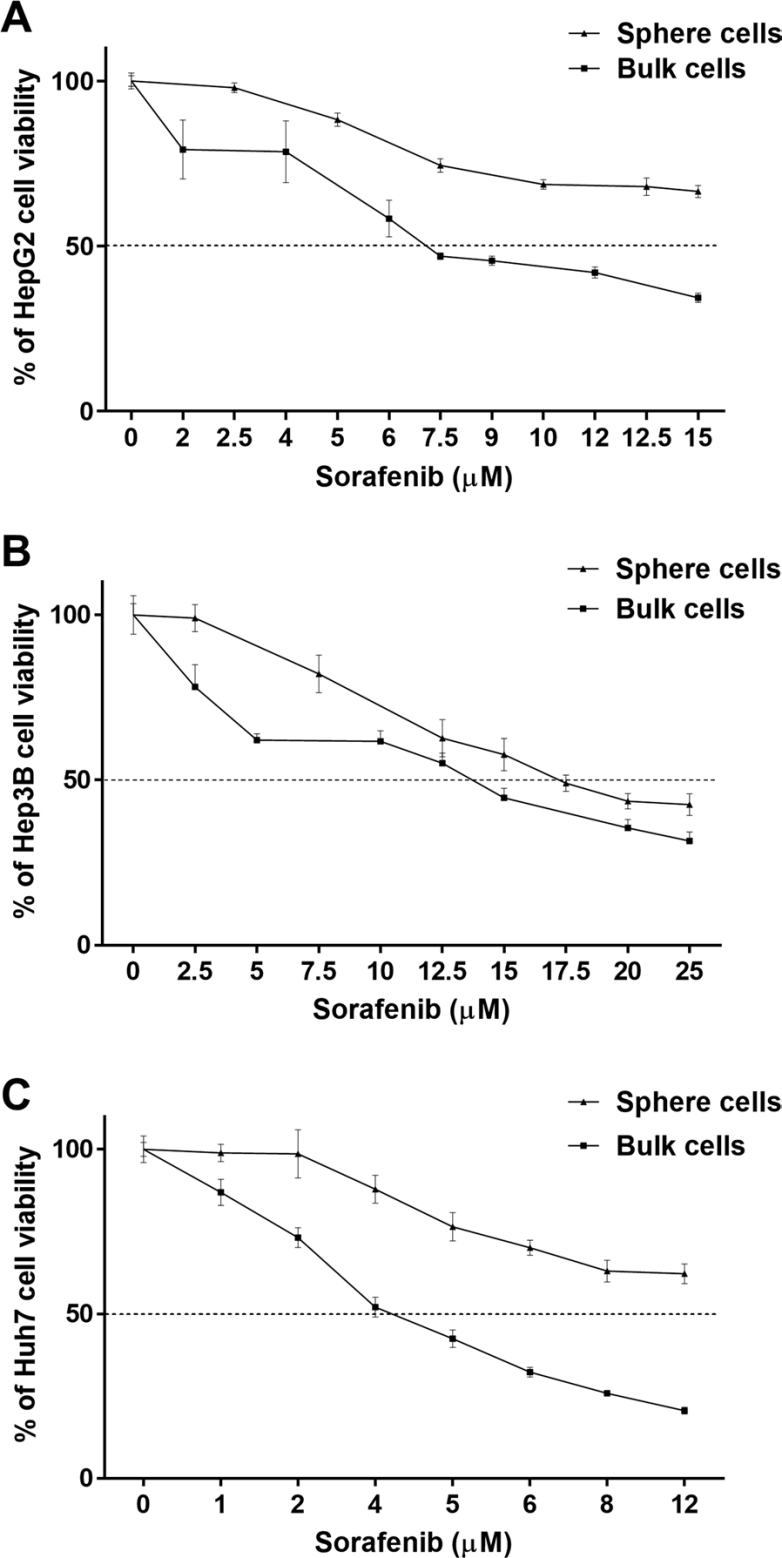
Cancer stem-like sphere cells enriched from HCC cell lines exhibit resistance to SOR. **A** HepG2 cells, **B** Hep3B cells, and **C** Huh7 cells were cultured in ultra-low attachment surface dishes for 14 days to form spheres. The spheres were dispersed into single cells to grow and were then treated with SOR. Cell viability was measured by MTT assay. The graphs represent the cytotoxicity profile of SOR at different concentrations as indicated on 48-h incubation

We next collected HCC sphere cells to assess their ability in resisting drug therapy. The IC_50_ doses of SOR for HepG2, Hep3B, and Huh7 sphere cells were > 15 μM (Fig. 4A), 17.1 μM (Fig. 4B), and 12 μM (Fig. 4C) respectively, higher than that for HepG2 bulk cells (7.1 μM), Hep3B bulk cells (13.7 μM), and Huh7 bulk cells (4.2 μM), revealing that CSC-enriched HCC spheres exhibit resistance to SOR. However, we also found that, in the presence of sorafenib, the viability of HepG2 sphere cells could be further increased by coculture with TAMs^CM^ or M2 Mφs^Cyto^ (Additional file 1: Fig. S3). This may be because 1) sphere cells are cells enriched for CSC properties but not real stem cells, and thus coculture with M2 TAMs can further boost the stemness of sphere cells, or 2) there are other mechanisms in addition to stemness induction utilized by M2 TAMs to promote SOR resistance of HCC cells.

### CXCL1 and CXCL2 released by M2 TAMs increase CSC stemness and reduce SOR-induced apoptosis

**Fig. 5:**
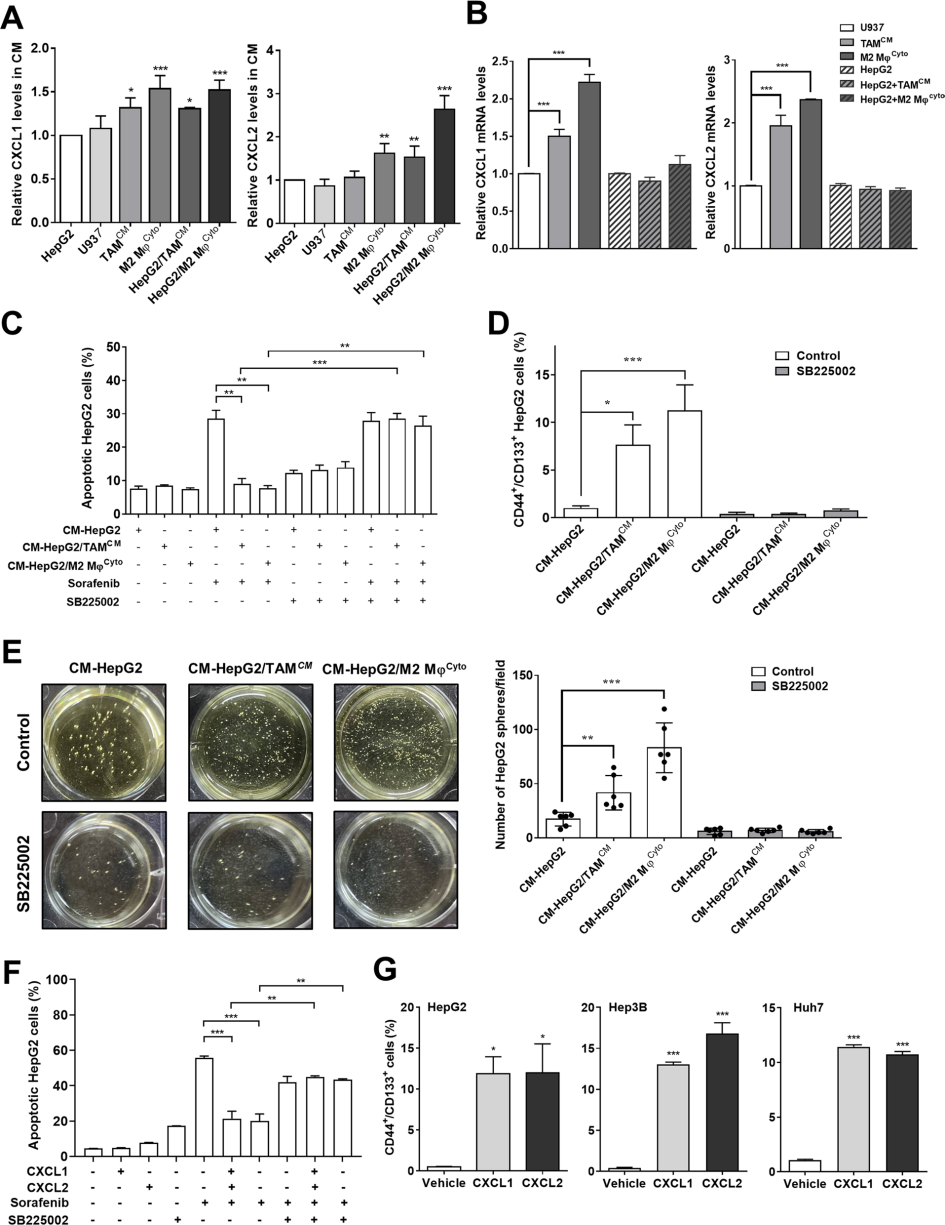
M2 TAMs release CXCL1 and CXCL2 to upregulate SOR resistance. **A** HepG2 cells, U937 cells, TAMs^CM^, and M2 Mφs^Cyto^ were monocultured for 72 h, and HepG2 cells were cocultured with TAMs^CM^ or M2 Mφs^Cyto^ for 72 h. The CM from monocultures and cocultures were collected for measurement of CXCL1 and CXCL2 levels using ELISA assays. The bar graphs depict the relative protein expression. *P < 0.05; **P < 0.01; ***P < 0.001 vs. HepG2. **B** CXCL1 and CXCL2 mRNA expression in U937 cells, TAMs^CM^, M2 Mφs^Cyto^, and HepG2 cells without or with 72-h coculture was measured by qPCR. The bar graphs show the relative mRNA expression. ***P < 0.001, significant difference between groups. **C** HepG2 cells were monocultured and cocultured with TAMsCM or M2 Mφs^Cyto^ for 72 h. The CM from monocultures and cocultures were collected to treat HepG2 cells in combination with SB225002 (50 μM) for 48 h followed by SOR (10 μM) for 48 h. The cells were collected to detect apoptosis using flow cytometry with double staining for Annexin V and PI. The bar graph shows the percentages of apoptotic cells. **P < 0.01; ***P < 0.001, significant difference between groups. **D** HepG2 cells were monocultured and cocultured with TAMsCM or M2 Mφs^Cyto^ for 72 h. The CM from monocultures and cocultures were collected to treat HepG2 cells in combination with SB225002 (50 μM) for 48 h followed by CSC identification. The bar graph shows the percentages of CD44^+^/CD133^+^ cells measured by flow cytometry with double staining for CD44 and CD133. *P < 0.05; ***P < 0.001 vs. CM-HepG2. **E** After treatment with different CM as indicated in combination with SB225002 (50 μM) for 48 h, HepG2 cells were collected and seeded (2000 cells/well) in ultra-low attachment surface dishes for 14 days to form spheres. The bar graph shows the numbers of spheres with a diameter greater than 100 μm in each field. **P < 0.01; ***P < 0.001 vs. CM-HepG2. **F** HepG2 cells were treated with CXCL1 (10 ng/mL) or CXCL2 (50 ng/mL) in combination with SB225002 (50 μM) for 18 h followed by SOR (10 μM) for 48 h. Apoptosis was assessed by flow cytometry with double staining for Annexin V and PI. The bar graph depicts the percentages of apoptotic cells. **P < 0.01; ***P < 0.001, significant difference between groups. **G** HepG2 cells, Hep3B cells, and Huh7 cells were treated with CXCL1 (10 ng/mL) or CXCL2 (50 ng/mL) for 18 h, and then cells were collected for flow cytometry with double staining for CD44 and CD133. The bar graphs show the percentages of CD44^+^/CD133^+^ cells. *P < 0.05; ***P < 0.001 vs. control

It has been shown that the secretion of several cytokines and chemokines such as IL-6, CXCL1, CXCL2, and CXCL5 was increased in the HCC cells/TAMs coculture medium [25, 33]. We measured IL-6, CXCL1, CXCL2, and CXCL5 levels in monoculture and coculture media and found that, as compared with HepG2 monoculture medium, only CXCL1 and CXCL2 were markedly upregulated in media of TAMs^CM^ and M2 Mφs^Cyto^ monocultures and HepG2 cells/TAMs^CM^ and HepG2 cells/M2 Mφs^Cyto^ cocultures (Fig. 5A). Because CXCL1 and CXCL2 have been reported to promote chemoresistance and CSC characteristics [7, 35], we thus sought to determine the importance of CXCL1 and CXCL2 in M2 TAMs-induced SOR resistance of HCC cells. Expression of CXCL1 and CXCL2 mRNA was significantly upregulated in TAMs^CM^ and M2 Mφs^Cyto^ compared with U937 cells but was not changed in HepG2 cells between with and without M2 TAMs coculture (Fig. 5B), suggesting that the increased secretion of CXCL1 and CXCL2 in coculture medium comes from M2 TAMs. Given that CXCL1 and CXCL2 exert their protumor function by activating the CXCR2 receptor, the selective and potent antagonist for CXCR2, SB225002 was used to determine the requirement of CXCR2 for M2 TAMs-mediated SOR resistance. Treatment with coculture media protected HepG2 cells from SOR-induced apoptosis, but this effect could be blocked by SB225002 (Fig. 5C). Similarly, coculture media-induced increases in CD44^+^/CD133^+^ cell number, sphere number, and stemness gene expression could also be reversed by SB225002 (Fig. 5D, E and Additional file 1: Fig. S4). To study the effects of CXCL1 and CXCL2 on SOR -induced cytotoxicity and the stemness, HCC cells were treated with recombinant CXCL1 and CXCL2. MTT assay to assess the viability of HepG2 cells pretreated with CXCL1 and CXCL2 at different concentrations followed by SOR treatment was performed, and we found that CXCL1 and CXCL2 could dose-dependently increase HepG2 cell survival in the presence of SOR, with the maximum effect observed at about 10–50 ng/mL and 50–100 ng/mL, respectively (Additional file 1: Fig. S5). Addition of CXCL1 or CXCL2 could suppress SOR-induced apoptosis in HepG2 cells (Fig. 5F), with increased gene expression of the antiapoptotic protein BCL-2 and decreased gene expression of the proapoptotic proteins BAD and BAX (Additional file 1: Fig. S6), and SB225002 could block the inhibitory effect of CXCL1 and CXCL2 on SOR-induced apoptosis (Fig. 5F). Addition of CXCL1 and CXCL2 could also increase the percentages of CD44^+^/CD133^+^ cells in HepG2, Hep3B, and Huh7 cells (Fig. 5G) and induce expression of the stemness-related genes *NANOG*, *SOX2*, *OCT4*, and *ALDHA1* (Additional file 1: Fig. S7). Collectively, these results demonstrate that CXCL1/2 and their receptor CXCR2 play key roles in M2 TAMs-induce SOR resistance and cancer stemness in HCC cells.

### Activation of ERK contributes to CXCL1/2-mediated resistance to SOR

**Fig. 6:**
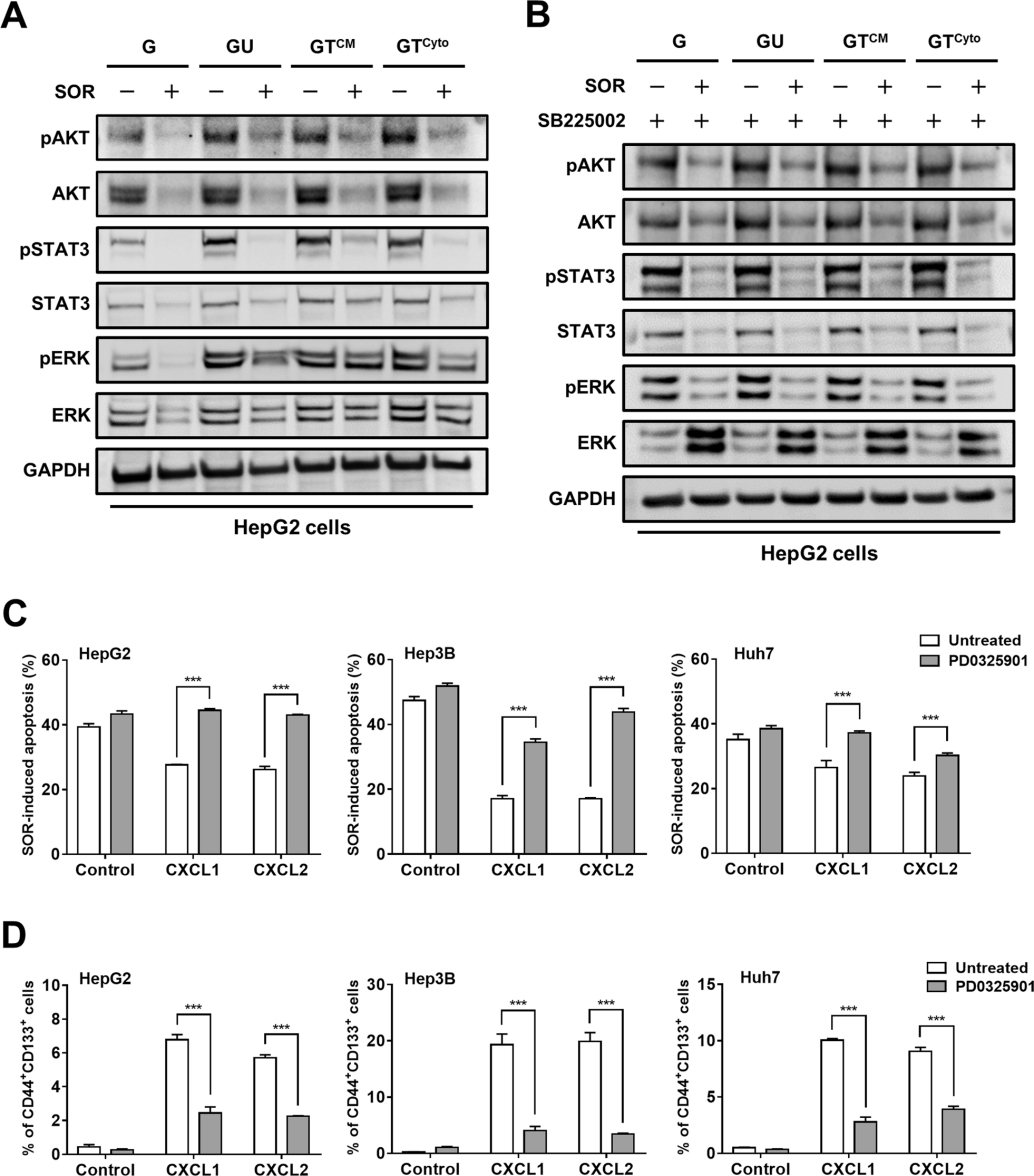
CXCL1 and CXCL2 promotes SOR resistance by activating the CXCR2/ERK pathway. **A** HepG2 cells were treated with CM and SOR (10 μM) for 48 h. Cell lysates were collected and subjected to Western blotting with the indicated antibodies. G: HepG2 monoculture CM, GU: HepG2/U937 coculture CM, GT^CM^: HepG2/TAM^CM^ coculture CM, GT^Cyto^: HepG2/ M2 Mφs^Cyto^ coculture CM. **B** HepG2 cells were treated with indicated CM, SB225002 (50 μM), and SOR (10 μM) for 48 h. Cell lysates were collected and subjected to Western blotting with the indicated antibodies. **C** HepG2 cells, Hep3B cells, and Huh7 cells were treated with CXCL1 or CXCL2 in combination with PD0325901 (10 μM) and SOR (10 μM for HepG2, 15 μM for Hep3B, 5 μM for Huh7) for 48 h. The bar graphs depict the percentages of apoptotic cells measured by flow cytometry with double staining for Annexin V and PI. ***P < 0.001 vs. untreated cells. **D** HepG2 cells, Hep3B cells, and Huh7 cells were treated with CXCL1 or CXCL2 in combination with PD0325901 (10 μM) for 24 h. The bar graphs depict the percentages of CD44^+^/CD133^+^ cells measured by flow cytometry with double staining for CD44 and CD133. ***P < 0.001 vs. untreated cells

SOR is a TKI with activity against multiple targets, including VEGFR, PDGFR and RAF kinases [36]. Activation of these signaling kinases leads to downstream signaling such as ERK, AKT, and STAT3. These pathways also act downstream CXCR2 after activation by CXCL1 and CXCL2 [10]. We found that SOR effectively suppress the phosphorylation of AKT and ERK in HepG2 cells as expected (Fig. 6A). Treatment with coculture CM induced the phosphorylation of AKT and ERK and prevented ERK phosphorylation from inhibition by SOR (Fig. 6A). Treatment with SOR in combination with SB225002 could almost completely suppress coculture CM-induced ERK phosphorylation (Fig. 6B), suggesting the phosphorylation of ERK by CXCR2 under SOR treatment. Pharmacological inhibition was further performed to determine the importance of ERK in CXCL1/2-mediated SOR resistance. CXCL1 and CXCL2 decreased SOR-induced apoptosis in HepG2 cells, Hep3B cells, and Huh7 cells, whereas treatment with PD0325901 to inhibit ERK could reverse this effect (Fig. 6C). Likewise, treatment with PD0325901 also blocked CXCL1- and CXCL2-mediated increased percentages of CD44^+^/CD133^+^ HCC cells (Fig. 6D). These data together suggest that M2 TAMs-derived CXCL1 and CXCL2 prevent HCC cells from SOR-induced cytotoxicity by activating the CXCR2/ERK pathway.

## Discussion

Despite advances in cancer care, conventional cancer treatments often fail to eradicate tumor cells because of metastasis, recurrence, heterogeneity, drug and radiation resistance, and evasion of immunological surveillance [30]. In recent years, a growing body of evidence supports the hypothesis that CSCs are a central cause responsible for treatment failure. CSCs are a unique subpopulations of cancer cells that display characteristics similar to normal stem or progenitor cells, such as self-renewal capability, stem cell signaling pathways, generation of progeny cells, resistance to chemotherapy and radiotherapy [12], and their existence has been confirmed in multiple cancer types, including HCC [18]. TAMs lie at the centre of the TME, affecting disease progression and outcome in cancer. It has been reported that TAMs enable cancer cells to acquire CSC-like properties [15]. In this study, we used in vitro coculture assays to demonstrate that M2 TAMs have ability to promote cancer stemness and interfere with the cytotoxic effect of SOR in HCC cells via paracrine signaling, suggesting that M2 TAMs act as a key driver in the development of drug resistance in HCC.

The roles for the chemokines CXCL1 and CXCL2 in tumor promotion has been mentioned in many studies, such as promoting epithelial-mesenchymal transition (EMT), invasiveness, metastasis, and cell survival under chemotherapy [1, 20]. Recently, the CXCL1 and CXCL2 pathways are also implicated in the generation of CSCs. For example, increased CXCL1 and PIGF expression promotes the expansion of lung CSCs, which in turn leads to the recurrence of lung cancer [23]. In colon cancer, the CXCL2-CXCR2 axis promotes tumorigenesis and contributes to CSC characteristics [7]. Consistently, we also identified the involvement of CXCL1 and CXCL2 in M2 TAM-induced SOR resistance and CSC properties of HCC cells. Pharmacological inhibition of CXCR2/ERK, the signaling pathway downstream of CXCL1 and CXCL2, could markedly decrease CSC activity and sensitize HCC cells to SOR. Interestingly, a recent report also showed that IL-8, CXCL1, and CXCL2 secreted by cancer-associated mesenchymal stromal cells can promote the polarization of TAMs [22]. These findings reveal the pivotal role for CXCL1 and CXCL2 in mediating formation and functions of M2 TAMs in HCC.

The Bcl-2 family consists of many protein members, which are divided into two groups with opposite effects on pro-apoptosis and anti-apoptosis. Thus, the mutual regulation between each other determines the trend of cell apoptosis [38]. Overwhelming evidence has shown that antiapoptotic BCL-2 is activated while proapoptic BAK and BAX are inhibited in many cancers, affecting the resistance of cancer cells to drugs [16]. In HCC, BAD is highly expressed and inhibition of its activity sensitizes HCC cells toward SOR-induced apoptosis, suggesting an important determinant of SOR response [17]. In addition, numerous recent studies have reported that CSCs in several types of cancer, such as leukemia, lung cancer, and colon cancer, exhibited overexpression of BCL-2 and BCL-XL that contributed to CSC survival [11, 21, 39]. The use of BH3 mimetics targeting the BCL-2 family eradicated CSCs and overcame drug resistance, suggesting that BCL-2 is an ideal target to eliminate both bulk tumor cells and CSCs. Our results showed that, besides CSC properties, CXCL1 and CXCL2 stimulated the transcription of *BCL-2* but inhibited the transcription of *BAD* and *BAX*, which might result in the resistance of CSC-enriched HCC sphere cells to SOR. This is supported by a previous report showing that CD133^+^ HCC cells exhibited higher survival rate and drug resistance in transplanted mouse models through activation of BCL-2 cell survival response [26].

## Conclusion

Through CXCL1 and CXCL2, M2 TAMs in the TME can promote the resistance of HCC cells to SOR. CXCR2/ERK is a key pathway participating in CXCL1- and CXCL2-mediated increase in CSC properties and BCL-2 expression. Targeting this signaling pathway to sensitize HCC cells to SOR and overcome the resistance mechanism may provide a novel method to improve tumor control and reduce the risk of HCC recurrence.

## Supplementary Information

**Additional file 1: Figure S1. **M2 polarization of U937 cell by coculture with HepG2 cells. U937 cells were cocultured with HepG2 cells for 72 h. U937 cells were then collected and subjected to flow cytometry with double staining for CD68 and CD204. **Figure S2. **Treatment with cytokines or coculture CM does not cause toxicity in U937 cells. **A** U937 cells were polarized towards TAMs^CM^ and M2 Mφs^Cyto^ by coculture CM and IL-4/IL-10, respectively, for 48 h. The nonadherent cells were collected for trypan blue assay and were visualized using bright field microscopy. Magnification: 100×. **B** U937 cells were incubated in normal RPMI medium or RPMI medium containing with 10% of coculture CM for 48 h. Total cells were collected for trypan blue assay, and an automated cell counter was used to count dead and live cells. The bar graphs depict the numbers of dead cells and live cells. NS, not significant vs. normal medium. **Figure S3. **Coculture with M2 TAMs further boosts sorafenib resistance of HepG2 sphere cells. HepG2 cells were cultured in ultra-low attachment surface dishes for 14 days to form spheres. The spheres were dispersed into single cells to monoculture and cocultured with TAMs^CM^ or M2 Mφs^Cyto^ for 72 h followed by SOR treatment at different concentrations for 48 h. Cell viability was determined by MTT assay. **Figure S4. **Pharmacological inhibition CXCR2 signaling reduces M2 TAMs-induced stemness. HepG2 cells were monocultured and cocultured with TAMs^CM^ or M2 Mφs^Cyto^ for 72 h. Conditioned media from monocultures and cocultures were collected to treat HepG2 cells in combination with SB225002 for 48 h followed by detecting mRNA expression of stemness genes including NANOG, SOX2, and OCT4 using qPCR. The bar graphs show relative mRNA expression of stemness genes. **, *P*<0.01; ***, *P*<0.001 vs. CM-HepG2. **Figure S5. **CXCL1 and CXCL2 protect HepG2 cells from sorafenib-induced cytotoxicity in a dose dependent manner. HepG2 cells were pretreated with CXCL1 or CXCL2 at different concentrations for 18 h followed by treatment with sorafenib for 48 h. MTT assay was performed to measure cell viability. **Figure S6. **CXCL1 and CXCL2 affect gene expression of BCL-2 family proteins in HCC cells after SOR treatment. **A** HepG2, **B** Hep3B, and **C** Huh7 cells were treated with CXCL1 (10 ng/μL) or CXCL2 (50 ng/μL) for 24 h followed by SOR (10 μM). Gene expression of BCL-2, BAD, and BAX was measured by qPCR. The bar graphs depict the relative mRNA expression. **P<0.01; ***P<0.001 vs. Control. **Figure S7. **CXCL1 and CXCL2 trigger expression of stem cell markers in HCC cells. **A** HepG2, **B** Hep3B, and **C** Huh7 cells were treated with CXCL1 (10 ng/μL) or CXCL2 (50 ng/μL) for 18 h. Gene expression of the stem cell markers NANOG, SOX2, OCT4, and ALDHA1 was determined by qPCR. The bar graphs depict the relative mRNA expression. **P<0.01; ***P<0.001 vs. Control.

## Data Availability

No available data and material.

## Abbreviations

SOR: Sorafenib
HCC: Hepatocellular carcinoma
TAM: Tumor associated macrophage
CSC: Cancer stem cell
TME: Tumor microenvironment
SNR: SOR nonresponse
SR: SOR response
TKI: Tyrosine kinase inhibitor
IF: Immunofluorescence
NCKUH: National Cheng Kung University Hospital
CM: Conditioned medium
RNAi: RNA interference
MTT: Colorimetric 3-(4,5-dimethylthiazol-2-yl)-2,5-diphe-nyltetrazolium bromide
qPCR: Quantitative polymerase chain reaction
