# Supplementary material for: Tumor-associated macrophages promote resistance of hepatocellular carcinoma cells against sorafenib by activating CXCR2 signaling
Source: J Biomed Sci. 2022 Nov 21;29:99. doi: 10.1186/s12929-022-00881-4 (PMC9677647; doi:10.1186/s12929-022-00881-4)
Supplement: Supplementary file 1 — Additional file 1: Figure S1. M2 polarization of U937 cell by coculture with HepG2 cells. U937 cells were cocultured with HepG2 cells for 72 h. U937 cells were then collected and subjected to flow cytometry with double staining for CD68 and CD204. Figure S2. Treatment with cytokines or coculture CM does not cause toxicity in U937 cells. A U937 cells were polarized towards TAMsCM and M2 MφsCyto by coculture CM and IL-4/IL-10, respectively, for 48 h. The nonadherent cells were collected for trypan blue assay and were visualized using bright field microscopy. Magnification: 100×. B U937 cells were incubated in normal RPMI medium or RPMI medium containing with 10% of coculture CM for 48 h. Total cells were collected for trypan blue assay, and an automated cell counter was used to count dead and live cells. The bar graphs depict the numbers of dead cells and live cells. NS, not significant vs. normal medium. Figure S3. Coculture with M2 TAMs further boosts sorafenib resistance of HepG2 sphere cells. HepG2 cells were cultured in ultra-low attachment surface dishes for 14 days to form spheres. The spheres were dispersed into single cells to monoculture and cocultured with TAMsCM or M2 MφsCyto for 72 h followed by SOR treatment at different concentrations for 48 h. Cell viability was determined by MTT assay. Figure S4. Pharmacological inhibition CXCR2 signaling reduces M2 TAMs-induced stemness. HepG2 cells were monocultured and cocultured with TAMsCM or M2 MφsCyto for 72 h. Conditioned media from monocultures and cocultures were collected to treat HepG2 cells in combination with SB225002 for 48 h followed by detecting mRNA expression of stemness genes including NANOG, SOX2, and OCT4 using qPCR. The bar graphs show relative mRNA expression of stemness genes. **, P<0.01; ***, P<0.001 vs. CM-HepG2. Figure S5. CXCL1 and CXCL2 protect HepG2 cells from sorafenib-induced cytotoxicity in a dose dependent manner. HepG2 cells were pretreated with CXCL1 or CXCL2 at different concentra [file 12929_2022_881_MOESM1_ESM.docx]

**Additional file 1**

**Tumor-associated macrophages promote resistance of hepatocellular carcinoma cells against sorafenib by activating CXCR2 signaling**

**Authors**:

Hao-Chen Wang^1^,

^1^Institute of Clinical Medicine, College of Medicine, National Cheng Kung University, Tainan, Taiwan, email: [esr2wang@gmail.com](mailto:esr2wang@gmail.com)

Lin-Ya Haung^1^,

^1^Institute of Clinical Medicine, College of Medicine, National Cheng Kung University, Tainan, Taiwan, email: [grace82822@gmail.com](mailto:grace82822@gmail.com)

Chih-Jung Wang^2^,

^2^Department of Surgery, National Cheng Kung University Hospital, College of Medicine, National Cheng Kung University, Tainan, Taiwan, email: [poemcage@gmail.com](mailto:poemcage@gmail.com)

Ya-Chin Hou^2^,

^2^Department of Surgery, National Cheng Kung University Hospital, College of Medicine, National Cheng Kung University, Tainan, Taiwan, email: [yachi2016@yahoo.com.tw](mailto:yachi2016@yahoo.com.tw)

Chia-Jui Yen^3^,

^3^Department of Oncology, National Cheng Kung University Hospital, College of Medicine, National Cheng Kung University, Tainan, Taiwan, email: [yencj@mail.ncku.edu.tw](mailto:yencj@mail.ncku.edu.tw)

Ying-Jui Chao^2^,

^2^Department of Surgery, National Cheng Kung University Hospital, College of Medicine, National Cheng Kung University, Tainan, Taiwan, email: [pitt_chao@yahoo.com.tw](mailto:pitt_chao@yahoo.com.tw)

Yan-Shen Shan^1, 2^,

^1^Institute of Clinical Medicine, College of Medicine, National Cheng Kung University, Tainan, Taiwan; ^2^Department of Surgery, National Cheng Kung University Hospital, College of Medicine, National Cheng Kung University, Tainan, Taiwan, Email: [ysshan@mail.ncku.edu.tw](mailto:ysshan@mail.ncku.edu.tw)

Corresponding Author:

Yan-Shen Shan, MD, PhD

Department of Surgery, National Cheng Kung University Hospital

Institute of Clinical Medicine, College of Medicine, National Cheng Kung University.

No. 35, Xiaodong Rd. Tainan City 704017, Taiwan

Tel: 886-6-2353535 ext. 3116

Fax: 886-6-2758781

Email: [ysshan@mail.ncku.edu.tw](mailto:ysshan@mail.ncku.edu.tw)


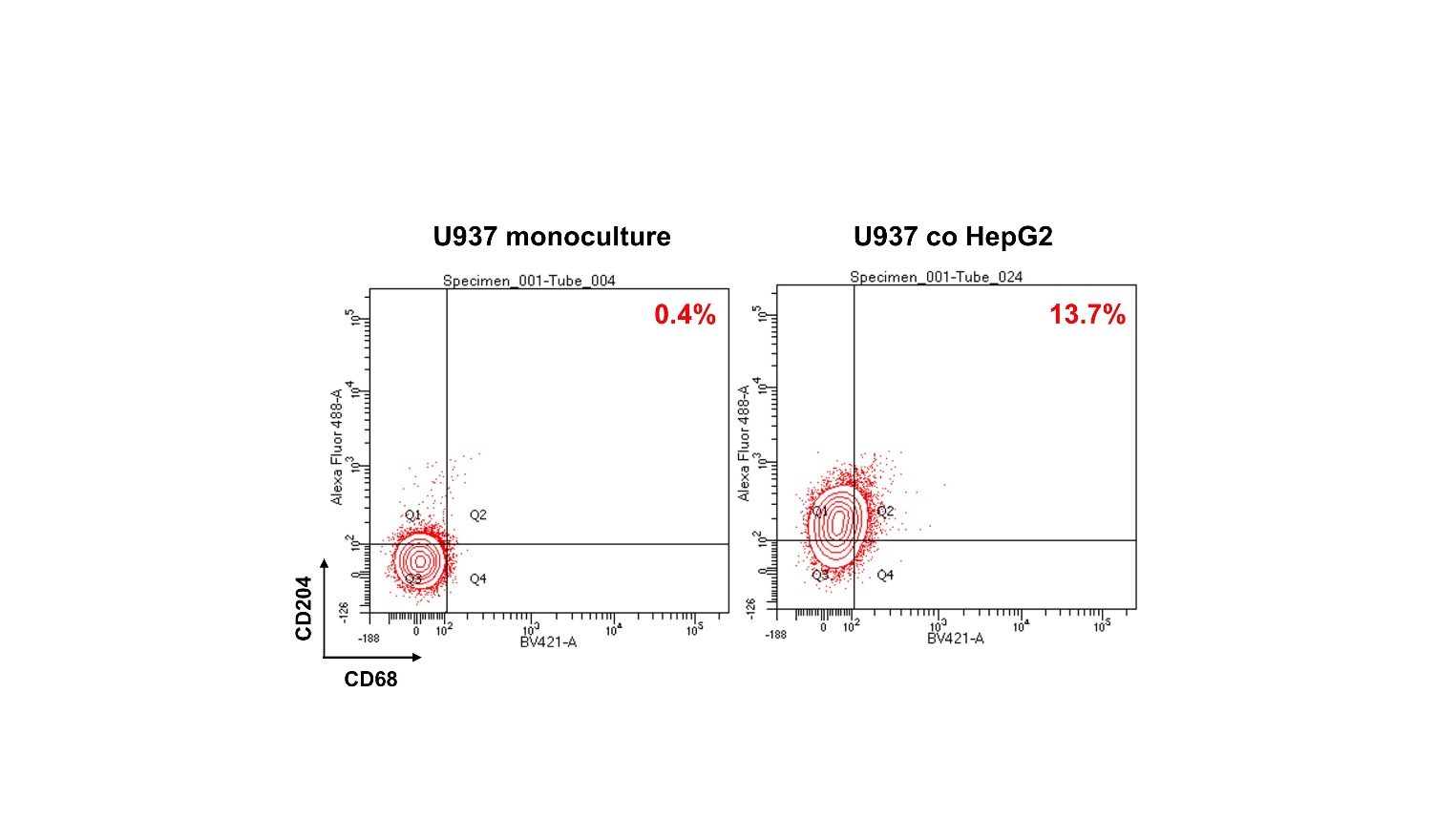


**Figure S1. M2 polarization of U937 cell by coculture with HepG2 cells.** U937 cells were cocultured with HepG2 cells for 72 h. U937 cells were then collected and subjected to flow cytometry with double staining for CD68 and CD204.


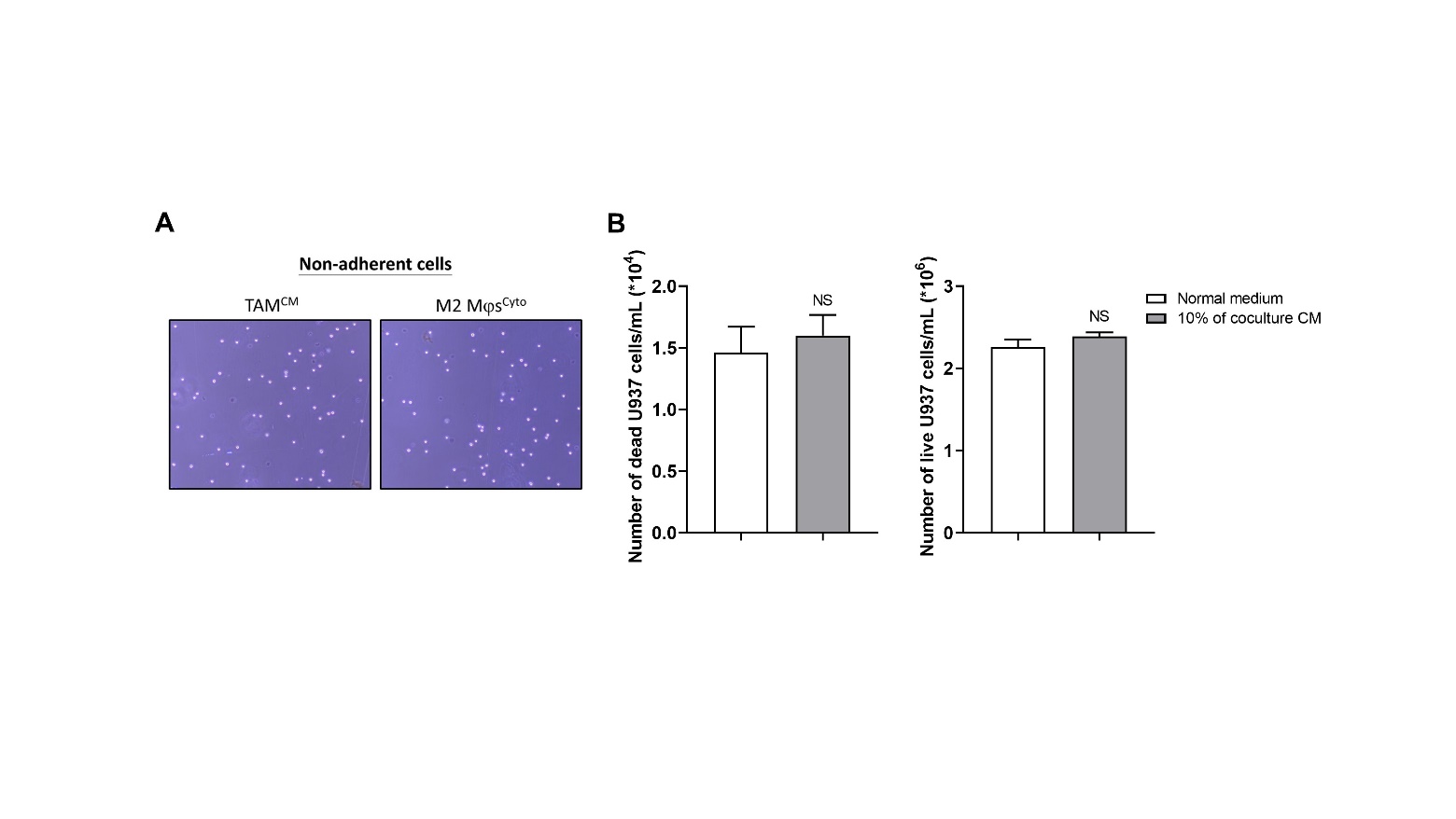


**Figure S2. Treatment with cytokines or coculture CM does not cause toxicity in U937 cells**. (A) U937 cells were polarized towards TAMs^CM^ and M2 Mϕs^Cyto^ by coculture CM and IL-4/IL-10, respectively, for 48 h. The nonadherent cells were collected for trypan blue assay and were visualized using bright field microscopy. Magnification: 100×. (B) U937 cells were incubated in normal RPMI medium or RPMI medium containing with 10% of coculture CM for 48 h. Total cells were collected for trypan blue assay, and an automated cell counter was used to count dead and live cells. The bar graphs depict the numbers of dead cells and live cells. NS, not significant versus normal medium.


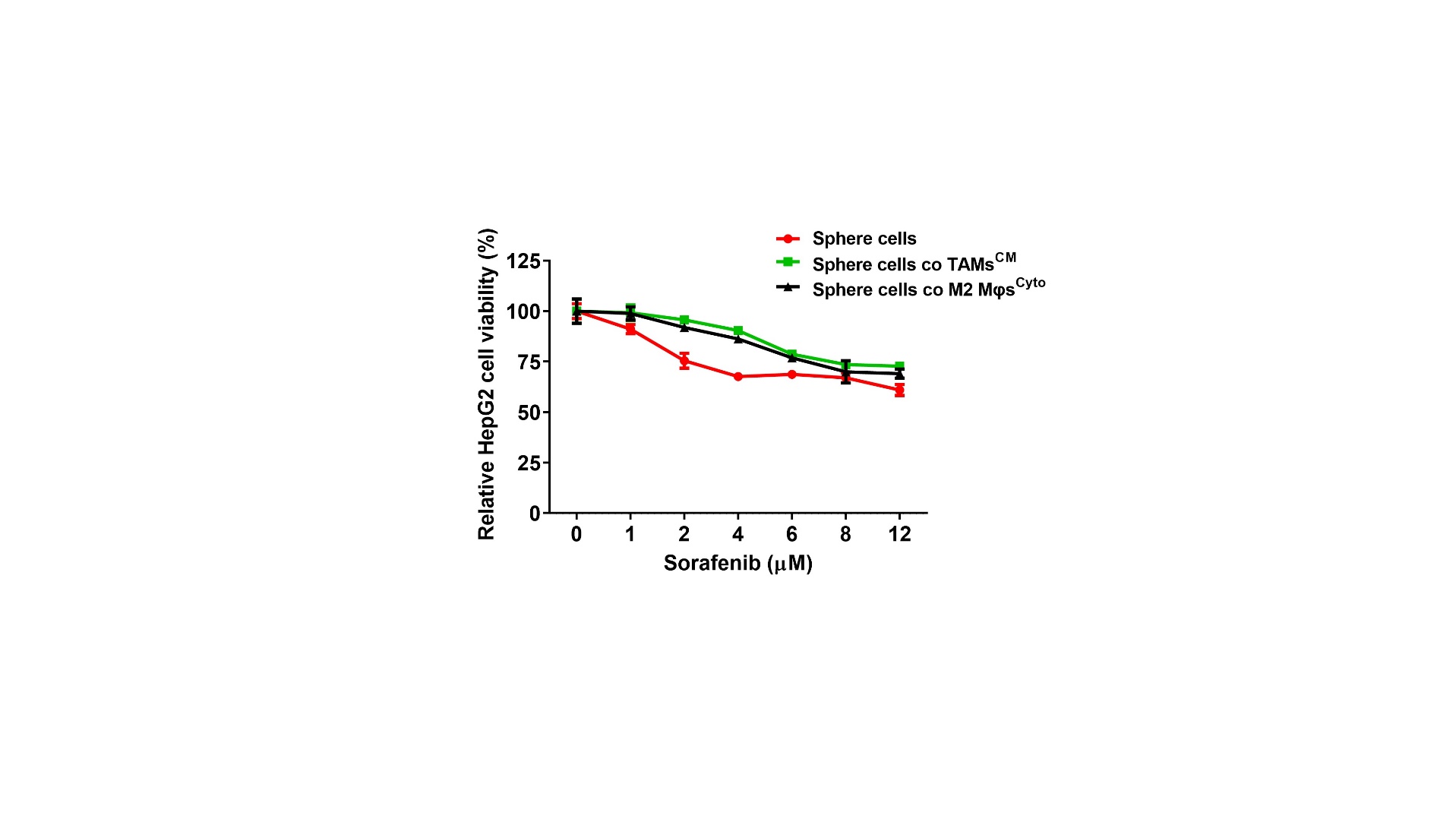


**Figure S3. Coculture with M2 TAMs further boosts** **sorafenib resistance of HepG2 sphere cells.** HepG2 cells were cultured in ultra-low attachment surface dishes for 14 days to form spheres. The spheres were dispersed into single cells to monoculture and cocultured with TAMs^CM^ or M2 Mϕs^Cyto^ for 72 h followed by SOR treatment at different concentrations for 48 h. Cell viability was determined by MTT assay..


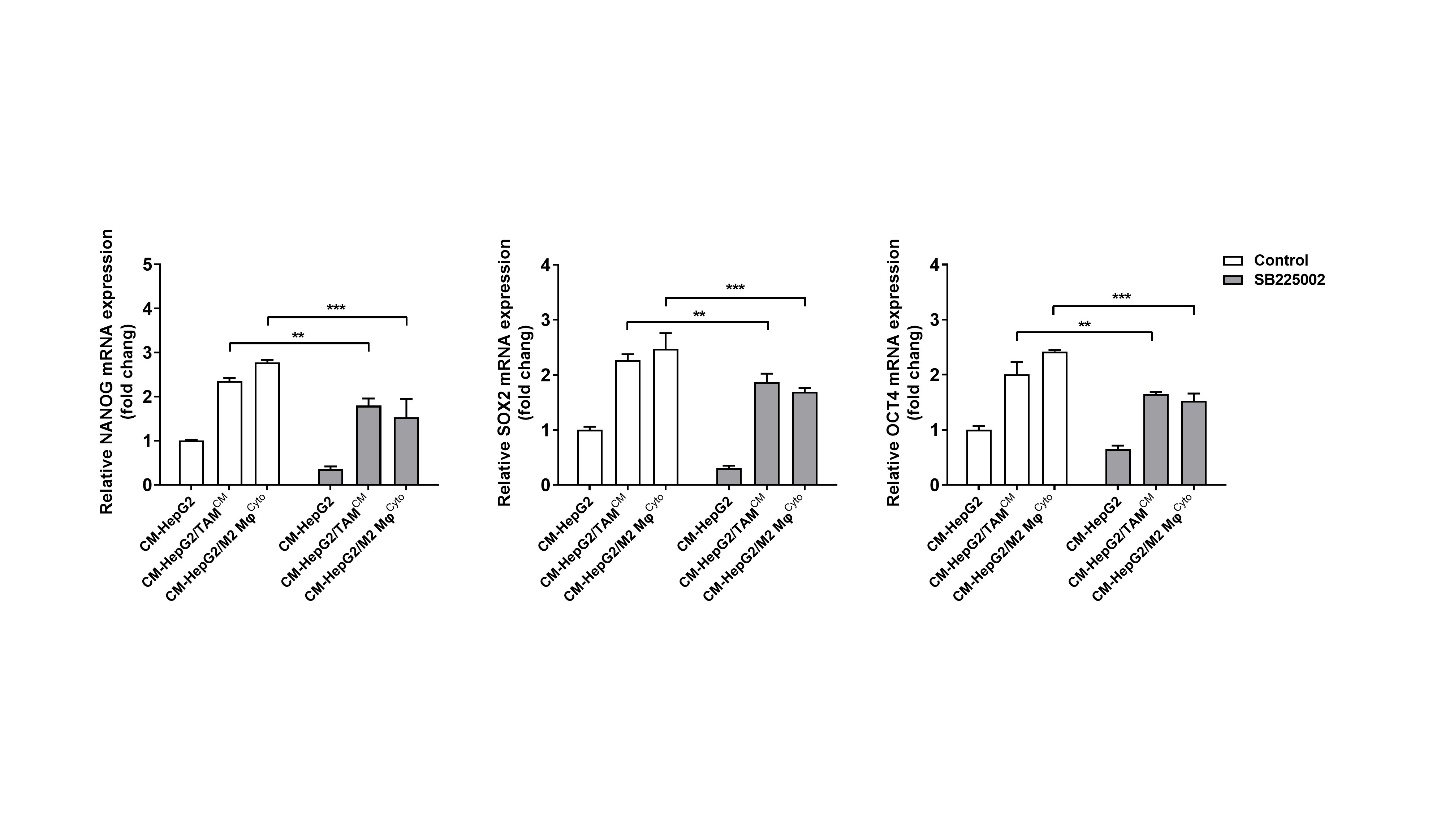


**Figure S4. Pharmacological inhibition CXCR2 signaling reduces M2 TAMs-induced stemness.** HepG2 cells were monocultured and cocultured with TAMs^CM^ or M2 Mϕs^Cyto^ for 72 h. Conditioned media from monocultures and cocultures were collected to treat HepG2 cells in combination with SB225002 for 48 h followed by detecting mRNA expression of stemness genes including NANOG, SOX2, and OCT4 using qPCR. The bar graphs show relative mRNA expression of stemness genes. **, *P*<0.01; ***, *P*<0.001 versus CM-HepG2.

**
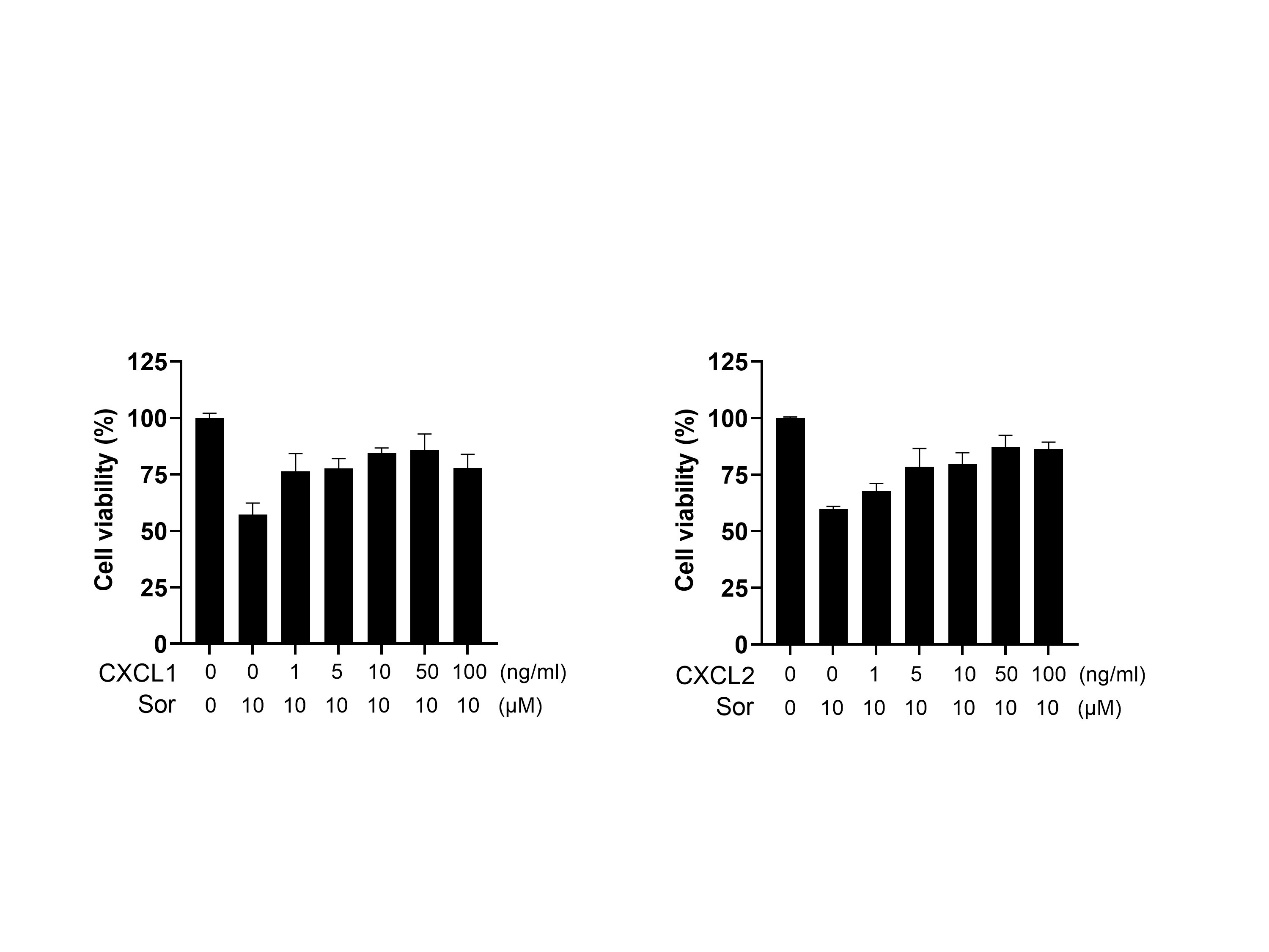
**

**Figure S5. CXCL1 and CXCL2 protect HepG2 cells from sorafenib-induced cytotoxicity in a dose dependent manner.** HepG2 cells were pretreated with CXCL1 or CXCL2 at different concentrations for 18 h followed by treatment with sorafenib for 48 h. MTT assay was performed to measure cell viability.

**
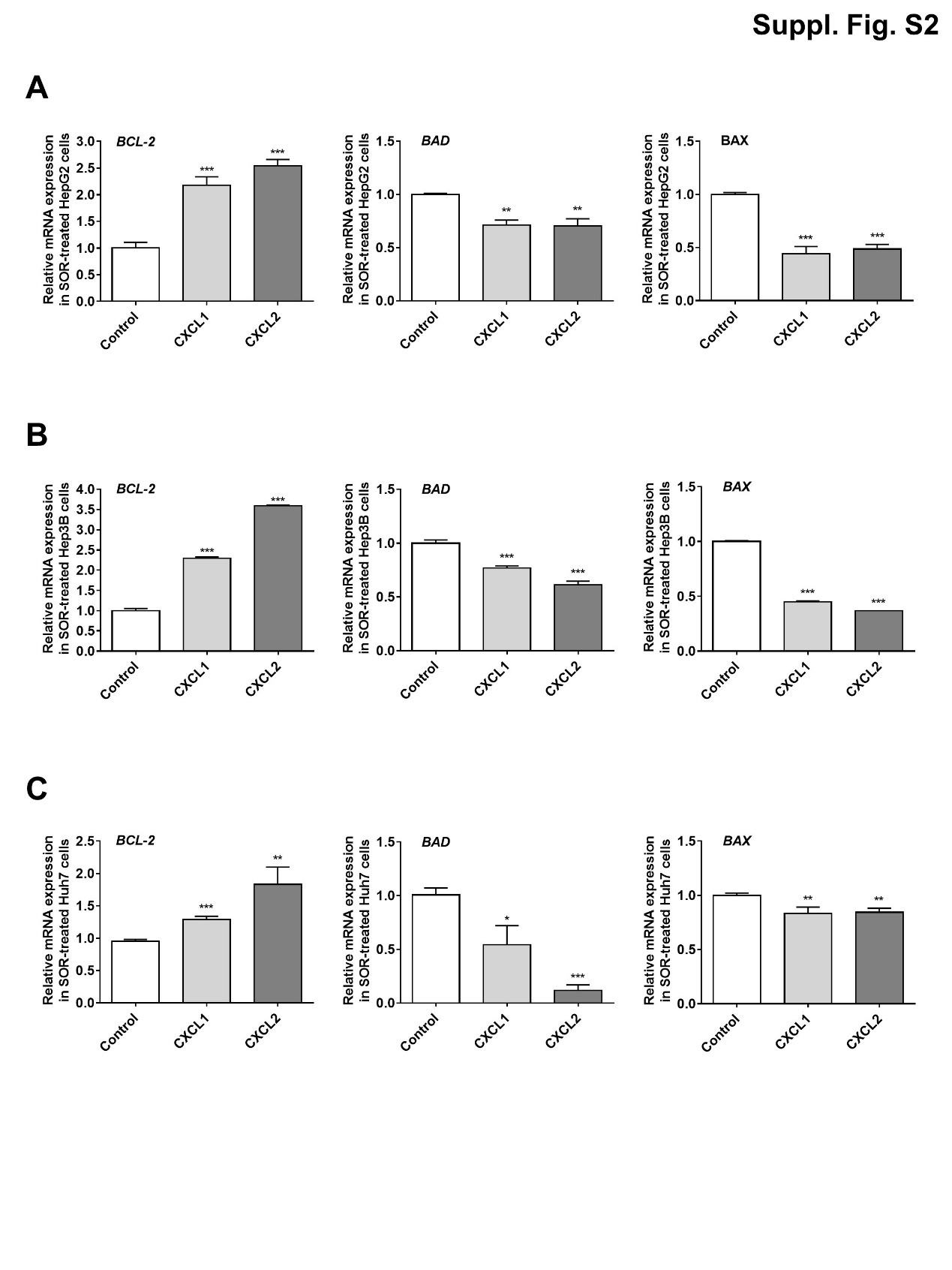
**

**Figure S6. CXCL1 and CXCL2 affect gene expression of BCL-2 family proteins in HCC cells after SOR treatment.** (A) HepG2, (B) Hep3B, and (C) Huh7 cells were treated with CXCL1 (10 ng/μL) or CXCL2 (50 ng/μL) for 24 h followed by SOR (10 μM). Gene expression of BCL-2, BAD, and BAX was measured by qPCR. The bar graphs depict the relative mRNA expression. **, *P*<0.01; ***, *P*<0.001 versus Control.

**
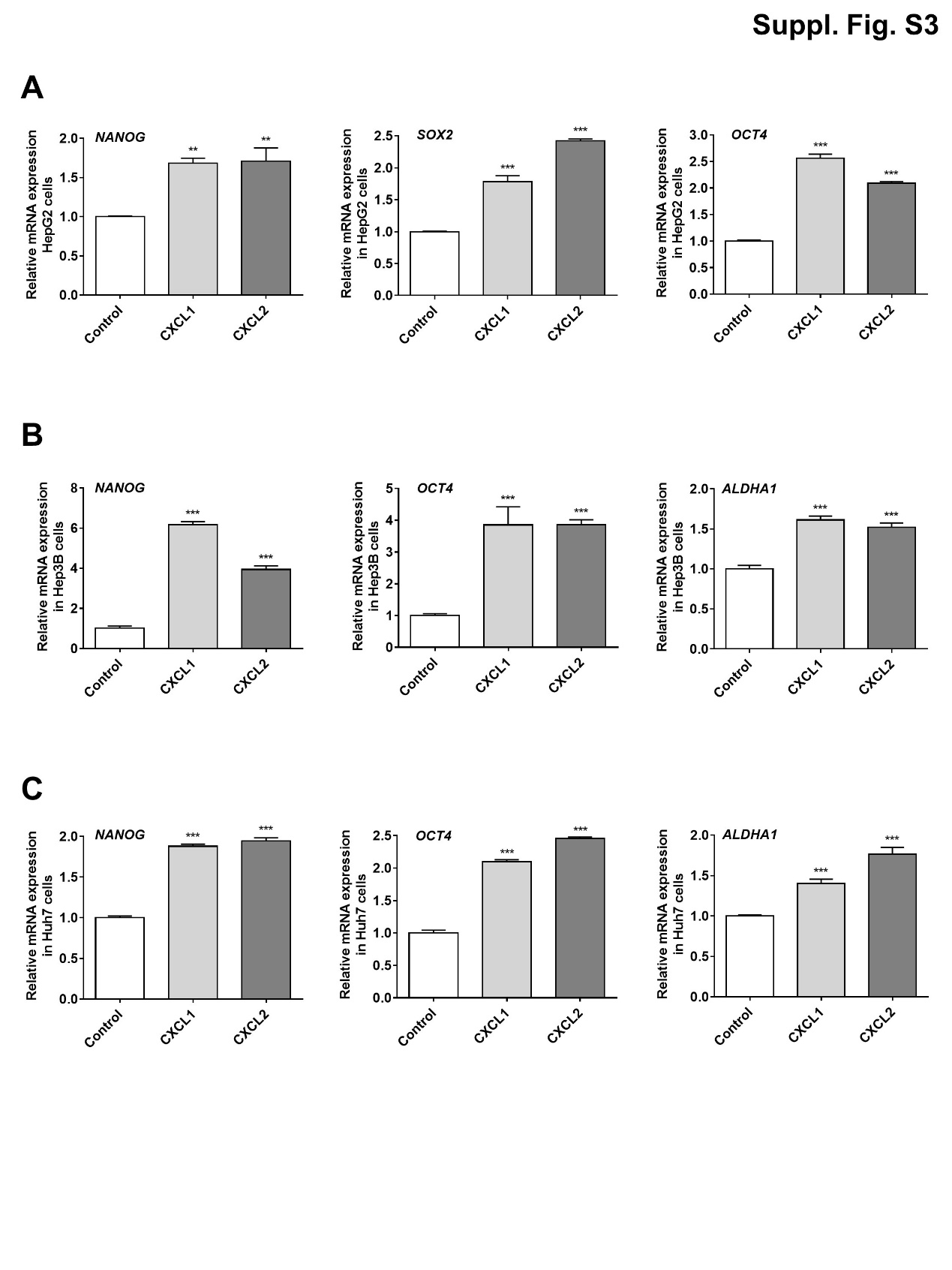
**

**Figure S7. CXCL1 and CXCL2 trigger expression of stem cell markers in HCC cells.** (A) HepG2, (B) Hep3B, and (C) Huh7 cells were treated with CXCL1 (10 ng/μL) or CXCL2 (50 ng/μL) for 18 h. Gene expression of the stem cell markers NANOG, SOX2, OCT4, and ALDHA1 was determined by qPCR. The bar graphs depict the relative mRNA expression. **, *P*<0.01; ***, *P*<0.001 versus Control.
